# Religiosity and Risk of Parkinson’s Disease in England and the USA

**DOI:** 10.1007/s10943-022-01603-8

**Published:** 2022-06-28

**Authors:** Abidemi I. Otaiku

**Affiliations:** 1 Department of Neurology, Birmingham City Hospital, Dudley Road, Birmingham, B18 7QH UK; 2 Centre for Human Brain Health, University of Birmingham, Birmingham, UK

**Keywords:** Parkinson’s disease, Religiosity, Spirituality, England, USA

## Abstract

Parkinson’s disease (PD) is associated with low religiosity cross-sectionally. Whether low religiosity might be associated with an increased risk for developing PD is unknown. This study investigated whether low religiosity in adulthood is associated with increased risk for developing PD. A population-based prospective cohort study was conducted. Participants from the English Longitudinal Study of Aging and the Midlife in the United States study who were free from PD at baseline (2004–2011) and completed questionnaires on self-reported religiosity, were included in a pooled analysis. Incident PD was based on self-report. Multivariable logistic regression was used to estimate odds ratios (OR) for developing PD according to baseline religiosity, with adjustment for sociodemographic characteristics, health and lifestyle factors and engagement in religious practices. Among 9,796 participants in the pooled dataset, 74 (0.8%) cases of incident PD were identified during a median follow-up of 8.1 years. In the fully adjusted model, compared with participants who considered religion very important in their lives at baseline, it was found that participants who considered religion “not at all important” in their lives had a tenfold risk of developing PD during follow-up (OR, 9.99; 95% CI 3.28–30.36). Moreover, there was a dose–response relationship between decreasing religiosity and increasing PD risk (*P* < 0.001 for trend). These associations were similar when adjusting for religious upbringing and when cases occurring within the first two years of follow-up were excluded from the analysis. The association was somewhat attenuated when religious practices were removed from the model as covariates, though it remained statistically significant (OR for “not at all important” vs. “very important”, 2.26; 95% CI 1.03–4.95) (*P* < 0.029 for trend). This longitudinal study provides evidence for the first time that low religiosity in adulthood may be a strong risk factor for developing PD.

## Introduction

The prevalence of Parkinson’s disease (PD) has increased substantially during the last three decades (GBD 2016 Parkinson's Disease Collaborators, 2018), with PD now representing the fastest growing neurological disorder in the world (GBD 2015 Neurological Disorders Collaborator Group, 2017). Moreover, with the aging of the world’s population, it has been predicted that the prevalence of PD could more than double within the next two decades (Dorsey et al., 2018). Given that there is currently no cure for PD, identifying risk factors for its development has now become a major public health priority (Ascherio & Schwarzschild., 2016).

Intriguingly, cross-sectional studies have consistently reported associations between PD and reduced intrinsic religiosity (Butler et al., 2010; Butler et al., 2011; Giaquinto et al., 2011; Kéri & Kelemen, 2016; McNamara et al., 2006), less frequent private religious practices (Butler et al., 2011; McNamara et al., 2006) and lower scores on measures of self-transcendence (Boussac et al., 2021; Pham et al., 2021) relative to age-matched controls.

Furthermore, despite people with PD being less likely to report having religious beliefs than matched controls (Giaquinto et al., 2011), they are however more likely than controls to report having spiritual beliefs (Giaquinto et al., 2011). Previous authors have speculated that PD may lead to a selective loss of religious faith in some patients (Butler & McNamara, 2016). However, recent neuroimaging research has raised the possibility that a lack of religious faith in some individuals could lead to PD (Ferguson et al., 2022).

Given that the prevalence of PD is increasing most rapidly among societies with a high proportion of religiously unaffiliated individuals (GBD 2016 Parkinson's Disease Collaborators, 2018; Pew Research Center's Religion & Public Life Project, 2018), and social science research has projected that religiosity will continue to decline in some parts of the world (Pew Research Center's Religion & Public Life Project, 2015)—it is clearly of high importance from a public health perspective, to clarify the temporal relationship between low religiosity and the development of PD. I hypothesised that low religiosity in adulthood would be associated with an increased risk for developing PD and tested this association using data from the English Longitudinal Study of Aging (ELSA) (Steptoe et al., 2013) and the Midlife in the United States (MIDUS) study (Radler, 2014).

## Methods

### Study Population

This longitudinal study used prospectively collected data from the English Longitudinal Study of Aging (ELSA) from July 2010 through to July 2019, and the Midlife in the United States (MIDUS) study from January 1995 through to June 2014. ELSA is an ongoing population-based cohort study which began in 2002, that has now included over 18,000 predominantly middle-aged and older adults who are representative of the English population (Steptoe et al., 2013). MIDUS is an ongoing population-based cohort study, beginning in 1995, which has included more than 7000 young, middle-aged and older adults from the USA, who were recruited through a nationally representative random-digit dialing sampling strategy, in addition to specific subsamples consisting of siblings and twins (Radler, 2014). Both studies set out to advance our understanding of the physical, psychological, social and economic changes associated with aging. Detailed descriptions of the two cohorts have been published elsewhere (Radler, 2014; Steptoe et al., 2013).

In ELSA, participants have been followed up approximately every 2 years since the baseline interview (“Wave 1”) and in MIDUS participants have been followed up every 9–10 years since the baseline survey (“MIDUS 1”). ELSA Wave 5 (2010–2011) and MIDUS 2 (2004–2006) were considered as the baseline for this analysis, since these were the first waves where information on religiosity, spirituality and PD were assessed. Follow-up for ELSA totaled 9 years and follow-up for MIDUS totaled 10 years.

To be included in this analysis, participants must have been free from PD at baseline and have completed all relevant questions on religion and spirituality (*n* = 11,644). Of these participants, those with missing data for any sociodemographic covariates (*n* = 166), or who did not take part in at least the first follow-up after baseline (*n* = 1682), were excluded. This yielded a total sample of 9796 participants, including 7124 (72.7%) participants from ELSA and 2672 (27.3%) participants from MIDUS.

## Measurements

### Religion and Spirituality

Religiosity was measured at baseline using the following question in ELSA: “How important is religion in your daily life?” (Berkessel et al., 2021) and a similar question in MIDUS: “How important is religion in your life?”. No definition of religion was provided to participants. The options for each question included: (1) very important, (2) somewhat important, (3) not very important, and (4) not at all important.

In a sensitivity analysis in MIDUS, participants were recategorised based on a combination of religiosity and spirituality. Spirituality was measured at baseline in MIDUS using the question: “How important is spirituality in your life?.” No definition of spirituality was provided. The response options were the same as those offered for religiosity. Participants could therefore be categorised into three groups: (a) “Religion very important”, (b) “Spirituality very important but not religion”, and (c) “Neither spirituality nor religion very important” (Vitorino et al., 2018).

Information on religious affiliation, frequency of religious/spiritual service attendance and frequency of private religious practices (prayer and meditation) were self-reported at baseline in both cohorts. Religious affiliations were recoded into three groups: (1) Christian religion, (2) non-Christian religion, and (3) no religion. Frequency of service attendance was recoded as: (1) more than weekly, (2) weekly, (3) monthly, (4) less than monthly, and (5) never. Frequency of private religious practices were recoded as: (1) daily, (2) often, (3) rarely, and (4) never.

Information on religious upbringing was assessed at MIDUS 1 (1995–1996), with the following question “How important was religion in your home when you were growing up?”. The options included: (1) very important, (2) somewhat important, (3) not very important, and (4) not at all important.

In a secondary analysis, participants in MIDUS were recategorised based on changes in religiosity prior to baseline. This was estimated by subtracting their scores for religiosity measured at baseline (MIDUS 2), from their scores for religiosity measured 10 years earlier (MIDUS 1). Participants could therefore be categorised into three groups based on pre-baseline changes in religiosity: (a) religiosity increased during the 10 years preceding baseline, (b) religiosity decreased during the 10 years preceding baseline, and (c) religiosity unchanged during the 10 years preceding baseline.

### Ascertainment of Incident PD

During the 10-year follow-up period, participants were asked at each interview (ELSA) or survey (MIDUS), to report whether they had ever been diagnosed with PD by a medical professional. Incident PD was defined as self-reported PD at the most recent interview/survey.

### Covariates

The following covariates, measured at baseline, were obtained by self-report in both cohorts: age in years (continuous), ethnicity (white, non-white), marital status (married, unmarried), educational qualifications (college degree or equivalent, high school or equivalent, none), smoking status (current, past, never), alcohol consumption (weekly, monthly, seldom/never), diabetes (yes/no), hypertension, (yes/no), mental health conditions (depression, anxiety, or other emotional problems, yes/no), self-rated health (good–excellent, poor/fair) and physical activity levels (light leisure or sporting activities, weekly, monthly, seldom/never). In addition, information on cognitive impairment (Alzheimer’s disease, other dementia, or serious memory impairment, yes/no) and severe mental disorders (bipolar disorder, schizophrenia, psychosis, yes/no) were available in ELSA. Information on head injuries (history of serious head injury, yes/no) was available in MIDUS. Missing values indicators were used for participants with missing information for these covariates.

### Statistical Analyses

The association of baseline religiosity with incident PD during follow-up was assessed using logistic regression to determine odds ratios (OR) with 95% confidence intervals (CI). The group that reported religion as being very important in their lives served as the reference. Religiosity was also entered as a single multilevel variable to test for a linear trend across groups.

Pooled and cohort-specific analyses were conducted with adjustment for possible confounders. Model 1 was minimally adjusted for age, sex and either cohort (pooled dataset), geographical region (ELSA), or sample (MIDUS). Model 2 additionally adjusted for ethnicity, education, marital status, smoking status, alcohol consumption, self-rated health, physical activity levels, diabetes, hypertension, mental health conditions, frequency of private religious practices, and frequency of religious service attendance.

Several sensitivity analyses were performed to confirm the robustness of the findings. Within the pooled dataset, the analyses were repeated after: (1) excluding participants who did not profess a religious affiliation at baseline, (2) restricting the analysis to participants with complete covariate data, and (3) removing religious practices as covariates in the regression model. In ELSA, the analyses were repeated after: (1) introducing a lag time of approximately 2 years, including only PD cases identified after the first visit, (2) excluding individuals with cognitive impairment or severe mental disorders at baseline, and (3) shortening the follow-up to the first 4 years (two visits) after baseline. In MIDUS, the analyses were repeated after: (1) adjusting for religious upbringing, (2) adjusting for a history of serious head injury at baseline, and (3) recategorising participants based on a combination of religiosity and spirituality.

In addition, a secondary analysis was carried out in MIDUS, which related changes in religiosity during the 10-years preceding baseline, with the subsequent risk of incident PD during follow-up.

Statistical testing was performed two-sided at *P* < 0.05. All analyses were performed using SPSS version 28 (IBM Corp., Armonk, NY).

## Results

### Study Cohorts

The baseline characteristics of the 7,124 participants in ELSA and 2,672 participants in MIDUS are presented in Tables 1 and 2, respectively. The mean (SD) baseline age was 65 (8.9) in ELSA and 55 (11.3) in MIDUS. Both cohorts had a higher proportion of women than men (ELSA: 55.4%; MIDUS: 55.8%). The participants were mostly white (ELSA: 97.2%; MIDUS: 93.1%) and predominantly reported a Christian religious affiliation (ELSA: 81.0%; MIDUS: 81.8%).

**Table 1:** Baseline characteristics of the ELSA study participants (*n* = 7124)

Variable	Religion Very Important (*n* = 1219)	Religion Somewhat Important (*n* = 1432)	Religion not Very Important (*n* = 2140)	Religion not at all Important (*n* = 2333)
Age (years)	67.9 ± 9.3	66.5 ± 8.9	65.3 ± 8.5	63.7 ± 8.5
Sex, *n* (%)				
Male	395 (32.4)	535 (37.4)	961 (44.9)	1283 (55.0)
Female	824 (67.6)	897 (62.6)	44.9 (55.1)	1050 (45.0)
Race, *n* (%)				
White	1099 (90.2)	1400 (97.8)	2119 (99.0)	2310 (99.0)
Non-white	120 (9.8)	32 (2.2)	21 (1.0)	23 (1.0)
Education, *n* (%)				
College degree	286 (23.5)	299 (20.9)	320 (17.3)	614 (26.3)
High school degree	683 (56.0)	823 (57.5)	1297 (60.6)	1263 (54.1)
No qualifications	250 (20.5)	310 (21.6)	473 (22.1)	456 (19.5)
Marital status, *n* (%)				
Married	799 (65.5)	988 (69.0)	1504 (70.3)	1601 (68.6)
Unmarried	420 (34.5)	444 (31.0)	636 (29.7)	732 (21.4)
Religious affiliation, *n* (%)				
Christian religion	1128 (92.5)	1385 (96.7)	2016 (94.2)	1243 (53.3)
Non-Christian religion	88 (7.2)	35 (2.4)	21 (1.0)	18 (0.8)
No religion	3 (0.2)	12 (0.8)	103 (4.8)	1072 (45.9)
Service attendance^a^	2.4 ± 1.3	3.7 ± 1.0	4.4 ± 0.6	4.7 ± 0.5
Prayer or meditation^b^	1.7 ± 0.9	2.6 ± 0.8	3.2 ± 0.7	3.6 ± 0.8
Health, *n* (%)				
Good–excellent	902 (74.0)	1105 (77.2)	1686 (78.8)	1846 (79.1)
Poor/fair	317 (26.0)	326 (22.8)	454 (21.2)	484 (20.7)
Missing	0 (0.0)	1 (0.1)	0 (0.0)	3 (0.1)
Mental health condition, *n* (%)				
Yes	112 (9.2)	143 (10.0)	182 (8.5)	214 (9.2)
No	1107 (90.8)	1288 (89.9)	1957 (91.4)	2117 (90.8)
Missing	0 (0.0)	1 (0.1)	1 (0.0)	2 (0.1)
Severe mental disorder, *n* (%)				
Yes	7 (0.6)	5 (0.3)	5 (0.2)	12 (0.5)
No	1212 (91.4)	1426 (99.6)	2134 (99.7)	2319 (99.4)
Missing	0 (0.0)	1 (0.1)	1 (0.0)	2 (0.1)
Cognitive impairment, *n* (%)				
Yes	5 (0.4)	12 (0.8)	12 (0.6)	16 (0.7)
No	1214 (91.6)	1420 (99.2)	2127 (99.4)	2317 (99.3)
Missing	0 (0.0)	0 (0.0)	1 (0.0)	0 (0.0)
Physical activity, *n* (%)				
Weekly	1093 (89.7)	1296 (90.5)	1941 (90.7)	2099 (90.0)
Monthly	20 (1.6)	35 (2.4)	60 (2.8)	73 (3.1)
Seldom/never	106 (8.7)	101 (7.1)	139 (6.5)	161 (6.9)
Hypertension, *n* (%)				
Yes	465 (38.1)	491 (34.3)	771 (36.0)	766 (32.8)
No	751 (61.6)	939 (65.6)	1363 (63.7)	1560 (66.9)
Missing	3 (0.2)	2 (0.1)	6 (0.3)	7 (0.3)
Diabetes, *n* (%)				
Yes	120 (9.8)	140 (9.8)	218 (10.2)	225 (9.6)
No	1099 (90.2)	1292 (90.2)	1921 (89.8)	2106 (90.3)
Missing	0 (0.0)	0 (0.0)	1 (0.0)	2 (0.1)
Alcohol consumption, *n* (%)				
Weekly	570 (46.8)	835 (58.3)	1360 (63.6)	1532 (65.7)
Monthly	149 (12.2)	196 (13.7)	227 (10.6)	260 (11.1)
Seldom/never	482 (39.5)	390 (27.2)	532 (25.1)	528 (22.6)
Missing	18 (1.5)	11 (0.8)	16 (0.7)	13 (0.6)
Smoking status, *n* (%)				
Current	78 (6.4)	153 (10.7)	261 (12.2)	361 (15.5)
Past	568 (46.6)	709 (49.5)	1069 (50.0)	1144 (49.0)
Never	573 (47.0)	470 (39.8)	810 (37.9)	828 (35.5)

Plus-minus values are means ± SD

^a^Scores range from 1 (more than weekly) to 5 (not at all)

^b^Scores range from 1 (daily) to 4 (never)

**Table 2:** Baseline characteristics of the MIDUS study participants (*n* = 2672)

Variable	Religion very important (*n* = 1094)	Religion somewhat important (*n* = 858)	Religion not very important (*n* = 488)	Religion not at all important (*n* = 232)
Age (years)	56.7 ± 11.5	53.9 ± 11.2	52.9 ± 11.0	52.9 ± 10.1
Sex, *n* (%)				
Male	391 (35.7)	397 (46.3)	258 (52.9)	134 (57.8)
Female	703 (64.3)	461 (53.7)	230 (47.1)	98 (42.2)
Race, *n* (%)				
White,	1003 (91.7)	801 (93.4)	461 (94.5)	223 (96.1)
Non-white	91 (8.3)	57 (6.6)	27 (5.5)	9 (3.9)
Education, *n* (%)				
College degree	522 (47.7)	420 (49.0)	276 (56.6)	155 (66.8)
High school degree	514 (47.0)	406 (47.3)	201 (41.2)	73 (31.5)
No qualifications	58 (5.3)	32 (3.7)	11 (2.3)	4 (1.7)
Marital status, *n* (%)				
Married	831 (76.0)	647 (75.4)	341 (69.9)	147 (63.4)
Unmarried	263 (24.0)	211 (24.6)	147 (30.1)	85 (36.6)
Religious affiliation, *n* (%)				
Christian religion	1061 (97.0)	770 (89.7)	312 (63.9)	43 (18.5)
Non-Christian religion	16 (1.5)	38 (4.4)	31 (6.4)	13 (5.6)
No religion	17 (1.6)	50 (5.8)	145 (29.7)	176 (75.9)
Service attendance^a^	2.1 ± 1.0	3.3 ± 1.2	4.3 ± 0.9	4.7 ± 0.9
Prayer or meditation^b^	1.3 ± 0.5	1.9 ± 0.8	2.7 ± 1.0	3.3 ± 1.0
Religious upbringing^c^	1.5 ± 0.7	1.8 ± 0.8	2.1 ± 0.9	2.5 ± 1.0
Spirituality^d^	1.2 ± 0.4	1.8 ± 0.6	2.3 ± 0.8	2.7 ± 1.2
Religious change^e^	0.3 ± 0.5	− 0.04 ± 0.7	− 0.3 ± 0.7	− 0.7 ± 0.7
Health, *n* (%)				
Good–excellent	980 (89.6)	775 (90.3)	451 (92.4)	209 (90.1)
Poor/fair	113 (10.3)	83 (9.7)	37 (7.6)	23 (9.9)
Missing	1 (0.1)	0 (0.0)	0 (0.0)	0 (0.0)
Mental health condition, *n* (%)				
Yes	195 (17.8)	142 (16.6)	92 (18.9)	48 (20.7)
No	899 (82.2)	716 (83.4)	396 (81.1)	184 (79.3)
Serious head injury, *n* (%)				
Yes	23 (2.1)	25 (2.9)	16 (3.3)	11 (4.7)
No	1071 (97.9)	832 (97.0)	472 (96.7)	221 (95.3)
Missing	0 (0.0)	1 (0.1)	0 (0.0)	0 (0.0)
Physical activity, *n* (%)				
Weekly	793 (72.5)	612 (72.0)	363 (74.4)	174 (75.0)
Monthly	190 (17.4)	163 (19.0)	96 (19.6)	40 (17.2)
Seldom/never	100 (9.1)	74 (8.6)	25 (5.1)	17 (7.3)
Missing	11 (1.0)	9 (1.0)	4 (0.8)	1 (0.4)
Hypertension, *n* (%)				
Yes	327 (29.9)	255 (29.7)	104 (21.3)	47 (20.3)
No	767 (70.1)	603 (70.3)	384 (78.7)	185 (79.7)
Diabetes, *n* (%)				
Yes	96 (8.8)	68 (7.9)	31 (6.4)	18 (7.8)
No	998 (91.2)	790 (92.1)	457 (93.6)	214 (92.2)
Alcohol consumption, *n* (%)				
Weekly	289 (26.4)	306 (35.7)	210 (43.0)	127 (54.7)
Monthly	282 (25.8)	266 (31.0)	125 (25.6)	41 (17.7)
Seldom/never	523 (47.8)	286 (33.3)	153 (31.4)	64 (27.6)
Smoking status, *n* (%)				
Current	103 (9.4)	130 (15.2)	78 (16.0)	38 (16.4)
Past	321 (29.3)	294 (34.3)	177 (36.3)	89 (38.4)
Never	670 (61.2)	434 (50.6)	233 (47.7)	105 (45.3)

Plus-minus values are means ± SD

Negative values indicate a decrease in religiosity during the 10-years preceding baseline

^a^Scores range from 1 (more than weekly) to 5 (not at all)

^b^Scores range from 1 (daily) to 4 (never)

^c^Scores range from 1 (very religious) to 4 (not at all religious)

^d^Scores range from 1 (spirituality very important) to 4 (spirituality not at all important)

^e^Scores range from − 3 to + 3. Positive values indicate an increase in religiosity during the 10-years preceding baseline

Overall, there were lower levels of religiosity in ELSA than in MIDUS. In ELSA, the largest group comprised participants who considered religion not at all important in their daily lives (32.7%) whilst the smallest group comprised participants who considered religion very important in their daily lives (17.1%). Whereas, in MIDUS, the largest group comprised participants who considered religion very important in their lives (40.9%) and the smallest group comprised participants who considered religion not at all important in their lives (8.7%).

### Baseline Religiosity and Risk of Developing PD

Among 9,796 participants in the pooled dataset, 74 cases of incident PD (0.8%) were identified during a median follow-up of 8.1 years (52 cases in ELSA, 22 cases in MIDUS). In the fully adjusted logistic regression model (Table 3), compared with participants who reported religion being very important in their lives at baseline, participants who reported religion being not at all important in their lives had a tenfold risk of developing PD in the pooled analysis (OR, 9.99; 95% CI 3.28–30.36). In addition, when religiosity was entered as a single multilevel variable to test for a linear trend across groups, there was a dose–response relationship between decreasing religiosity and increasing PD risk (*P* < 0.001 for trend). These associations were also significant in both cohort-specific analyses (Table 3).

**Table 3:** Baseline religiosity and odds ratios (95% CI) for incident Parkinson’s disease

Study, analysis	Religion very important	Religion somewhat important	Religion not very important	Religion not at all important	*P* value for trend
*Pooled*					
PD cases [*n* (%)]	11 (0.5)	16 (0.7)	25 (1.0)	22 (0.9)	
*N*	2313	2290	2628	2565	
Model 1	1 [reference]	1.62 (0.75, 3.53)	2.44 (1.16, 5.13)*	2.34 (1.07, 5.11)*	0.021*
Model 2	1 [reference]	2.90 (1.20, 7.00)*	7.82 (2.85, 21.47)***	9.99 (3.28, 30.36)***	< 0.001***
*ELSA*					
PD cases [*n* (%)]	5 (0.4)	6 (0.4)	21 (1.0)	20 (0.9)	
*N*	1219	1432	2140	2333	
Model 1	1 [reference]	1.08 (0.33, 3.57)	2.60 (0.96, 6.99)	2.23 (0.82, 6.08)	0.047*
Model 2	1 [reference]	1.29 (0.34, 4.91)	5.83 (1.37, 24.83)*	6.89 (1.48, 32.01)*	0.006**
*MIDUS*					
PD cases [*n* (%)]	6 (0.5)	10 (1.2)	4 (0.8)	2 (0.9)	
*N*	1094	858	488	232	
Model 1	1 [reference]	2.30 (0.81, 6.51)	1.68 (0.45, 6.21)	1.74 (0.33, 9.10)	0.406
Model 2	1 [reference]	5.53 (1.67, 18.32)**	8.42 (1.68, 42.10)**	13.69 (1.63, 114.97)*	0.003**

ELSA, English Longitudinal Study of Aging; MIDUS, Midlife in the United States study; PD, Parkinson’s disease

Model 1: age, sex and either cohort (pooled), region (ELSA), or sample (MIDUS)

Model 2: Model 1 + ethnicity, education, marital status, smoking status, alcohol consumption, physical activity levels, self-rated health, diabetes, hypertension, mental health conditions, frequency of private religious practices, and frequency of religious service attendance

^*^P < 0.05

^**^P < 0.01

^***^P < 0.001

### Sensitivity Analyses

Within the pooled dataset, lower baseline religiosity was associated with a higher risk of developing PD even when restricting the analysis to participants who professed a religious affiliation (*P* < 0.001 for trend). Compared with religiously affiliated individuals who reported religion being very important in their lives at baseline, religiously affiliated individuals who reported religion being not at all important in their lives had a greater than tenfold risk of developing PD (OR, 10.46; 95% CI 3.12–35.07). Complete case analysis (*n* = 9684) revealed a similar association (OR, 9.19; 95% CI 3.00–28.17). The associations were somewhat attenuated when removing religious service attendance (OR, 4.03; 95% CI 1.62–10.04), private religious practices (OR, 6.84; 95% CI 2.36–19.78), or both religious service attendance and private religious practices (OR, 2.26; 95% CI 1.03–4.95) from the regression model, though they remained statistically significant. Furthermore, there was still a linear trend across the groups when all religious practices were removed from the model (*P* < 0.029 for trend).

In ELSA, the association remained significant after excluding incident PD cases that were diagnosed within the first 2 years of follow-up (OR, 5.18; 95% CI 1.02–26.44), and after excluding individuals with cognitive impairment or severe mental disorders at baseline (OR, 6.88; 95% CI 1.49–31.85). The association was considerably strengthened after shortening the follow-up to the first 4 years after baseline (OR, 18.14; 95% CI 1.82–180.49).

In MIDUS, the association was strengthened after adjusting for religious upbringing (OR, 17.39; 95% CI 2.00–151.0) and was unchanged after adjusting for a history of serious head injury (OR, 12.95; 95% CI 1.54–108.85). After recategorising participants based on a combination of religiosity and spirituality, participants who considered spirituality very important in their lives but not religion (OR, 7.68; 95% CI 2.21–26.63) and participants who considered neither spirituality nor religion very important in their lives (OR, 4.25; 95% CI 1.06–17.06), were both at higher risk of developing PD compared to participants who considered religion very important.

### Change in Religiosity and Risk of Developing PD

After recategorising participants in MIDUS based on changes in religiosity during the 10-years preceding baseline, compared with participants whose level of religiosity was unchanged during this period, participants whose level of religiosity decreased had a higher risk of developing PD (OR, 3.31; 95% CI 1.16–9.49), whereas participants whose level of religiosity increased had a lower risk of developing PD (OR, 0.29; 95% CI 0.04–2.36).

## Discussion

Using prospective data from two population-based cohort studies in England and the USA, the current study shows for the first time that low religiosity in adulthood may be associated with an increased risk for developing PD, accounting for a wide range of potential confounders.

The findings of this longitudinal study are consistent with previous cross-sectional studies, which showed a robust association between PD and low religiosity (Boussac et al., 2021; Butler et al., 2010; Butler et al., 2011; Giaquinto et al., 2011; Kéri & Kelemen, 2016; McNamara et al., 2006; Pham et al., 2021), case-reports showing improvement of parkinsonism after intense religious experiences (Moreno & de Yebenes, 2009) and theoretical work, that has offered biologically plausible mechanisms by which religiosity could confer neuroprotection in PD (Yulug et al., 2015). The results are also in keeping with a recent neuroimaging study (Ferguson et al., 2022), which showed that brain lesions causing parkinsonism, intersect brain regions associated with religiosity.

It is noteworthy that participants who considered spirituality very important in their lives but not religion, had a higher risk for developing PD than participants who considered religion very important, and also participants who considered neither spirituality nor religion very important. This finding is consistent with an earlier study, which showed that individuals with PD, though less likely to have religious beliefs than matched controls, are on the other hand more likely than controls to have spiritual beliefs (Giaquinto et al., 2011). As such, this study corroborates previous research which suggests that individuals who have a spiritual understanding of life in the absence of a religious framework, may be more vulnerable to developing neuropsychiatric disorders (King et al., 2013; Vitorino et al., 2018).

These results are also in agreement with previous studies, which found higher religiosity to be associated with lower risk of developing a wide range of physical (Ahrenfeldt et al., 2017, 2019; Li et al., 2016), mental (Edlund et al., 2010; Miller et al., 2012; Opsahl et al., 2019) and cognitive disorders (Lin et al., 2015). However, the magnitude of the association found in this study is considerably higher than for any physical health condition previously reported, and therefore requires explanation. A recent study identified that individuals with high self-reported intrinsic religiosity may have significantly higher levels of brain-derived neurotrophic factor (BDNF) than individuals with low self-reported intrinsic religiosity (Mosqueiro et al., 2019). Given that BDNF has been shown to enhance the survival of dopaminergic neurons in animal models of PD (Palasz et al., 2020) and BDNF levels are significantly reduced in patients diagnosed with PD (Jiang et al., 2019), it is plausible that differences in BDNF levels among healthy adults with different levels of religiosity, could partially explain the dose–response relationship with PD risk observed in this study. In addition, there is accumulating evidence that dopaminergic pathways play a central role in mediating religious experience (Previc, 2006; van Elk & Aleman., 2017). A recent SPECT study found significant changes in dopamine transporter binding in the basal ganglia after attendance at a one-week Christian retreat (Newberg et al., 2018). Earlier studies showed increased dopamine release in the ventral striatum during certain forms of meditation (Kjaer, et al., 2002) and increased blood flow to the caudate nucleus during silent religious prayer (Schjødt et al., 2008). These studies suggest that habitual engagement in religious activities could modify dopamine levels in brain regions linked to PD pathology. Therefore, given strong preclinical evidence that enhancing dopamine neurotransmission with dopamine agonists confers neuroprotection in PD (Schapira & Olanow, 2003); it is plausible that individuals with higher religiosity, also have higher midbrain dopamine levels, and consequently have more protection against developing PD.

It is important to note however, that these results do not necessarily imply that religious participation should now be promoted by public health agencies as a preventative measure for PD; given that people’s religious beliefs and commitments are highly personal, and are not usually arrived at based on health concerns. Moreover, further studies are still required to confirm the exact biological mechanisms linking lower religiosity and PD.

Also, seemingly in contrast to the present findings, previous studies have repeatedly shown that clergy and religious workers—who are presumably high in religiosity—have a higher risk for developing PD compared to adults in the general population (Park et al., 2005; Schulte et al., 1996; Tanner et al., 2009). Although, this association is attenuated when the total number of years having worked in a religious occupation is adjusted for (Tanner et al., 2009). The most parsimonious explanation for this observation, would be that the increased risk for PD is confined to individuals with a religious occupation who subsequently experience a decline in religiosity. However, this suggestion is speculative and future studies will be required to confirm this hypothesis.

In addition, future studies are warranted to determine which aspects of religiosity are most associated with the risk of PD, especially given the striking change in the estimates when religious practices (particularly religious service attendance) were included as covariates in this analysis. On the surface, this would seem to imply that religious practices were harmful, i.e., participants with higher religiosity had a lower risk of developing PD *despite* engaging in more frequent religious practices. However, this would contradict the previously mentioned literature which seems to suggest that religious practices might be protective. Alternatively, it is possible that participants who engaged in more frequent religious practices, but considered religion relatively unimportant in their daily lives, may have exhibited low intrinsic religiosity—but high extrinsic religiosity. If so, it may be the case that having high extrinsic religiosity in the presence of low intrinsic religiosity, is an even stronger risk factor for developing PD than having consistently low religiosity (i.e., low intrinsic and extrinsic religiosity). Accordingly, adjusting for religious practices might have made the association more apparent—by isolating the effects of intrinsic religiosity on PD. Intriguingly, this theory may be in line with a recent cross-sectional study, which showed that newly diagnosed people with PD had lower intrinsic religiosity than age-and sex- matched healthy controls, despite the two groups being similar for frequency of religious practices (Kéri & Kelemen, 2016). Thus, if this theory is confirmed to be true, this might further explain why some clergy and religious workers are at higher risk of developing PD.

## Strengths and Limitations

This study has several strengths, including the prospective design, long follow-up period, use of two large and well-documented population-representative cohorts, inclusion of a wide range of potential confounders, measurement of religiosity at two different time periods in two different continents and employment of a variety of sensitivity analyses. Furthermore, the participants were not selected on the basis of religiosity or PD diagnosis. Several limitations also warrant discussion. Following previous published studies (Kamel et al., 2007; Leng et al., 2018, 2020) this study relied on self-reporting to determine incident PD and therefore may have missed or misclassified some cases. Second, the small number of cases within each level of religiosity led to wide confidence intervals. It is also difficult to fully exclude the possibility of reverse causality, as low religiosity might be an early sign of undiagnosed PD, rather than a risk factor for developing PD (given that PD often has a long latency from motor symptom onset to diagnosis) (Breen et al., 2013). However, the long follow-up period coupled with the findings from the 2-year time lag analysis, suggest that low religiosity preceded the development of clinical PD. This would also be consistent with a recent longitudinal study, which showed that PD does not cause religiosity to decline (Redfern et al., 2020). Moreover, the analysis using 10-year changes in religiosity showed that becoming more religious over time reduced the subsequent risk of developing PD, which implies that low religiosity may cause PD. Previous studies have shown that PD patients with symptoms beginning on the left-side of their body, are less religious on average than PD patients whose symptoms begin on their right-side (Butler et al., 2011; Giaquinto et al., 2011). As information on PD characteristics were not available in this study, it was not possible to confirm whether individuals with low religiosity were more likely to develop left-onset PD. Finally, the findings from this study might not be generalizable to predominantly non-Christian populations (Lin et al., 2015).

## Conclusions

This study provides evidence for the first time that low religiosity in adulthood may be associated with an increased risk for developing PD; especially among individuals who have a spiritual, but not religious, outlook on life. If replicated by other researchers, these findings could prove important in understanding global trends in the incidence of PD (GBD 2016 Parkinson's Disease Collaborators, 2018). Furthermore, these findings may help to improve the early detection of PD and may stimulate new approaches for delaying or preventing disease onset.

## Data Availability

Data from ELSA are available by application through the UK Data Service: http://doi.org/10.5255/UKDA-SN-5050-23. Data from MIDUS are available by application through the National Archive of Computerized Data on Aging: https://www.icpsr.umich.edu/web/NACDA/series/203.
